# Supporting the Manufacturing of Medical Supplies in Africa: Collaboration Between Africa CDC, Partners, and Member States

**DOI:** 10.9745/GHSP-D-23-00121

**Published:** 2023-10-30

**Authors:** Abdulaziz Mohammed, Hanna Idris-Dantata, Tochi Okwor, Paul Tanui, Elijah Paintsil, Patrick Chanda Kabwe, Yewande Alimi, Raji Tajudeen, Wessam Mankoula, Olayinka Stephen Ilesanmi, Muhammad Shakir Balogun, Chikwe Ihekweazu, Emilio Hornsey, Onyema Ogbuagu

**Affiliations:** a Africa Centres for Disease Control and Prevention, Addis Ababa, Ethiopia.; b Health Maxima, LLC, Gaithersburg, MD, USA.; c Nigeria Centres for Disease Control and Prevention, Abuja, Nigeria.; d African Union Development Agency-NEPAD, Midrand, South Africa.; e Yale School of Medicine, Yale University, New Haven, CT, USA.; f African Field Epidemiology Network, Kampala, Uganda.; g UK Health Security Agency, London School of Hygiene and Tropical Medicine, London, United Kingdom.

## Abstract

Inadequate supply of PPE, vaccines, and diagnostics during the COVID-19 pandemic in Africa created an opportunity to promote local manufacturing. Authors describe Africa CDC's contributions and highlight strategies for strengthening the pandemic response.

## Key Messages

- Inadequate supply of personal protective equipment, vaccines, diagnostics, and therapeutics during the COVID-19 pandemic in Africa created an opportunity to promote local manufacturing.
- South Africa, Morocco, Tunisia, Egypt, and Senegal are currently involved in local vaccine production that meets approximately 1% of the total continental vaccine demand and should rank topmost in capacity-building for increased production.
- Four concepts—cooperation, collaboration, coordination, and communication—are underlined in the Africa Centres for Disease Control and Prevention's strategy to promote the procurement of large supplies of diagnostics, therapeutics, and other essential supplies.
- By investing in existing manufacturers, challenges in market access can be overcome. Local manufacturers in South Africa, Morocco, Tunisia, Egypt, and Senegal are able to sell to Gavi, the Vaccine Alliance-supported countries and can achieve volume certainty at scale.
- Beyond manufacturing facilities, specialized financing is needed to improve larger enablers such as expertise for manufacturing plants and research institutions, legal frameworks for vaccine approval, and research and development, particularly for novel vaccines.

## INTRODUCTION

The Africa Centres for Disease Control and Prevention (Africa CDC) is an autonomous body of the African Union (AU) established to support public health initiatives of member states.^1^ It aims to strengthen the capacity of Africa's public health institutions to detect, prevent, control, and respond quickly and effectively to disease threats.^1^^–^^3^ To promote pandemic preparedness and outbreak response, Africa CDC, its partners, and member states have collaborated on noteworthy steps to support local manufacturing of vaccines, personal protective equipment (PPE), diagnostics, and therapeutics in Africa.^3^^–^^5^ These goals are central to the mission of the New Public Health Order, a roadmap that strives to strengthen the resilience of health systems across the region.^6^ In this commentary, we describe the collaborations between Africa CDC, member states, and partners in boosting the production of PPE, vaccines, and diagnostics in Africa and highlight activities to be prioritized.

The detrimental impact of the ongoing COVID-19 pandemic across Africa's health, social, and economic sectors necessitated a special event—“Africa's New Public Health Order: Rejuvenating the Global Health Security Agenda”—held on the sidelines of the 77th Session of the United Nations General Assembly.^6^ One of the New Public Health Order's 5 pillars is the expansion of local manufacturing of health products. All vaccine purchasing mechanisms, such as Gavi, the Vaccine Alliance and other stakeholders, are requested to purchase at least 30% of their vaccines from manufacturers in Africa.^7^ In addition, governments, multilateral organizations, and civil society organizations have been requested to remove barriers related to trade and intellectual property to support local production of health products within the region.

## COOPERATION, COLLABORATION, COORDINATION, AND COMMUNICATION DURING THE PANDEMIC RESPONSE

Four concepts—cooperation, collaboration, coordination, and communication—are underlined in the Africa CDC's strategy and the New Public Health Order.^8^

### Cooperation

The Africa Task Force for Novel Coronavirus was established by the Africa CDC on February 4, 2020, shortly before the first case of COVID-19 was discovered in Africa, to organize the pandemic response throughout the continent.^8^ All 55 of the continent's ministers of health attended an emergency conference called by Africa CDC to examine the COVID-19 pandemic and agree on a continental plan.^8^ To avoid overloading the already overworked health care institutions across the continent, this strategy centered on limiting transmission and minimizing community spread.^9^ Such continental leadership has ensured a coordinated response to the pandemic and given direction to member states.

### Collaboration

This continental strategy was combined with regional cooperation, of which collaboration within the East African Community is a good example. African nations' top priority was to halt the spread of the virus; thus, the East African Community invested in the development of a regional electronic cargo and driver tracking system to monitor COVID-19 cases as they crossed international borders. The use of truck traffic across borders to transport necessities like medication enabled Rwanda to digitally communicate the COVID-19 test results of the truck drivers.^8^^,^^9^

Without other preventive measures, the adoption of border closure in African countries had little impact on the occurrence of COVID-19.^10^ While many Western nations delayed closing borders, the majority of African nations handled border closure seriously to protect their citizens.^9^ To complement the preventative plan, lockdowns and border closures were put into place as soon as the first few cases were reported on March 15, and flights were prohibited.^9^ Most African countries also promptly adopted other prevention interventions, such as the use of face masks, handwashing, and social distancing.^10^

### Coordination

The African Union Commission and the Africa CDC launched the Partnership to Accelerate COVID-19 Testing (PACT) strategy to facilitate implementation of the Africa Joint Continental Strategy for COVID-19 that was endorsed by African ministers of health and approved by the Bureau of the Assembly of the African Union Heads of State and Government in March 2020.^8^ PACT focused on the establishment of warehousing and distribution hubs across Africa, in partnership with organizations including the World Food Programme and Ethiopian Airlines. The warehousing model has been used to successfully distribute medical supplies and donations from the Jack Ma Foundation Initiative. Efforts are ongoing on the rehabilitation of the warehouse at Yaoundé, Cameroon.

The PACT strategy focused on coordinating pooled procurement of diagnostics and other commodities for distribution across the continent and mobilizing support for deploying 1 million community health care workers for contact tracing.^8^ As part of the goal for economic recovery and reopening, the strategy placed a high priority on data analysis and prompt dissemination of results on technological platforms to increase public confidence in testing data, epidemiological models, and essential health forecasting methods.

The PACT strategy focused on coordinating pooled procurement of diagnostics and other commodities for distribution across the continent and mobilizing support for deploying 1 million community health care workers for contact tracing.

### Communication

Many African countries used social media channels and other traditional media sources, such as radio, to improve community sensitization about COVID-19 and its preventive measures. This open communication channel and adequate community involvement boosted the community's trust in the public health system and contributed to the public's adherence to prevention and response guidelines.^8^ The Rwandan government used robots to measure individuals' temperature in public settings and health facilities. Likewise, they used drones for mass communication, surveillance, and medicine delivery.

## ACTORS AND STAKEHOLDERS IN THE COVID-19 RESPONSE

### Pandemic Response

#### Jack Ma Foundation

During the early stages of the COVID-19 pandemic, Africa CDC, in collaboration with the Jack Ma Foundation, organized online seminars and training sessions for public health officials.^11^^,^^12^ Four webinar series were conducted (in English and French), each of which comprised 6 weekly sessions.^11^ These online sessions took place during April–December 2020 and catered to participants from various AU countries. The English webinars trained participants from 48 AU countries, while the French webinars trained participants from 37 AU countries.

To ensure wider accessibility, all webinars were recorded and made available on the Zoom cloud and YouTube. By December 31, 2020, the recordings had amassed a total of 3,616 views.^11^ Additionally, the webinars were streamed live on social media platforms, such as Facebook, which attracted a total of 5,630 viewers. Among them, 2,539 watched the English webinars, while 3,091 tuned in for the French webinars.^11^ Health workers who watched the webinars were encouraged to conduct additional trainings for their colleagues at their primary workplaces.

#### Susan Thompson Buffett Foundation

With the aid of a grant from the Susan Thompson Buffett Foundation that is on sexual and reproductive health services, Africa CDC prioritized the needs of vulnerable groups as core elements of essential health services.^13^ To enhance health outcomes during the COVID-19 pandemic, it was imperative to focus on increasing accessibility to maternal and child health services. One of the achievements of the partnership with the foundation was the purchase and distribution of medical supplies to health care facilities providing maternal and child care and sexual reproductive health services in 10 of the member states.^13^

#### Jack Ma and Ali Baba Foundations

During the COVID-19 pandemic, the Jack Ma and Ali Baba Foundations provided consignment of medical equipment in 3 phases.^14^ These included nearly 2 million test kits, swabs, ventilators, and infection control materials.^14^

#### Mastercard Foundation

The Mastercard Foundation and the Africa CDC have partnered on the 3-year, US$1.5 billion Saving Lives and Livelihoods initiative.^15^ This initiative aims to speed up Africa's economic recovery after the COVID-19 pandemic by saving the lives and livelihoods of millions of people. More than 65 million people are receiving immunizations because of the Saving Lives and Livelihoods initiative.^15^ The partnership between Mastercard Foundation and Africa CDC extends beyond the acute phase of the pandemic, with goals to integrate COVID-19 vaccination into the health care system and strengthen primary health care in Africa.

### Vaccine Production

The goal of the vaccine manufacturing plan for Africa is to ensure prompt access to vaccines for safeguarding public health by creating a support system for vaccine development and manufacturing within Africa. A key target is to locally produce 60% of Africa's regular immunization requirements by 2040, in line with the New Public Health Order.^6^^,^^16^ Many actors have been involved in the realization of this goal.

The goal of the vaccine manufacturing plan for Africa is to ensure prompt access to vaccines for safeguarding public health by creating a support system for vaccine development and manufacturing within Africa.

#### Saving Lives and Livelihood Initiative

In addition to its work during the acute phase of the pandemic, the partnership has also paved the way for vaccine production in Africa and strengthened the manufacturing of human vaccines.^15^

#### African Development Bank

Through a flagship program, the African Development Bank supports local vaccine manufacturing in line with its 2030 Vision for the Development of Africa's Pharmaceutical Industry and the 2040 AU/Africa CDC vision for increased local vaccine manufacturing.^17^ The Bank's interventions are part of a strategic pillar to increase the maturity of the industry through the development of local production capacities and 4 enablers, including (1) enabling regional logistic integration, (2) supporting the implementation of quality industry standards, (3) seeding the creation of research and development capacities, and (4) paving the way for increased vaccine manufacturing.^17^

#### Afreximbank

Afreximbank replicated its successful collaboration with the AU and Africa CDC during the COVID-19 pandemic to support the vaccine manufacturing platform at various stages of the vaccine manufacturing value chain.^17^ This has been achieved through early-stage project preparatory support, financial advisory services, project finance, and risk-bearing instruments.

### Manufacturing and Standardization of PPE

#### African Medical Supplies Platform

The Africa CDC, the Economic Commission for Africa, Afreximbank, and AU Special Envoy Strive Masiyiwa collaborated to create the African Medical Supplies Platform (AMSP).^18^ AMSP was intended to last beyond the pandemic, though it was established in the context of the opportunities and challenges of COVID-19. The purpose of the platform was to address supply chain issues such as shortages, delays in distributing supplies, accessibility, and affordability.^18^ The sector of the African economy that manufactures medical supplies is growing partly due to AMSP.^19^

#### Africa Medical Devices Forum and the Africa Medical Regulatory Harmonization Consortium

The Africa Medical Devices Forum and the Africa Medical Regulatory Harmonization consortium are major continent-level regulatory agencies that are being enhanced to address regulations and standards for the manufacturing of PPE.^20^ The vision of the consortium was to strengthen institutional regulatory capacity, provide programs with a single set of guidelines for transparency with regulatory processes and clear timelines, and enable resource pooling, including twinning programs, with the aim of expediting approval of medical products.^20^ The consortium has engaged 55 national medicines regulatory agencies across the continent but found variability in national approaches to medicines and devices regulation.^21^ Hence, while efforts are geared toward strengthening individual country regulatory capacity, the long-term goal is to establish a continent-wide regulatory agency, the Africa Medicines Agency. The creation of the Africa Medicines Agency will promote the implementation of a harmonized regulatory framework for regulation of medical devices and in vitro diagnostics, as well as support training and capacity development in the Africa region.^21^ The South Africa Bureau of Standards assures the quality of critical products, including their safety and reliability.^22^ Cross-country peer-to-peer exchange learning systems have also been established, where countries with limited capacity are supported to understudy countries equipped with capacity in accreditation and regulations, which facilitates knowledge transfer and adoption of best practices.^22^ The structure and process used by United Nations agencies for medical device standardization, including post-market surveillance, have also been initiated.^22^ Based on the existing diverse country-level experience and varying degrees of PPE standards, an essential framework for the standard testing of PPE has been defined.^22^

#### UNICEF

As a stopgap measure, African countries have collaborated with UNICEF for quality assurance to promote safe and efficient production of PPE, which has helped the continent conform to ISO standards for quality management systems in the provision of medical devices.^23^ Additionally, predelivery inspection and certification have been scaled up to foster quality assurance.^23^ Predelivery inspection is carried out at the supplier's premises and is a requirement before shipping goods. Certification is a conformity assessment process whereby an independent neutral body confirms that a product or service complies with relevant specifications or standards.^23^^,^^24^

## FRAMEWORKS GUIDING AFRICA CDC MEDICAL SUPPLIES

### Framework for the Standard Testing of PPE

The Standard Testing of PPE Framework outlines the guidelines and protocols developed by Africa CDC for the rigorous evaluation and assessment of PPE.^5^^,^^10^ This framework provides a standardized approach for testing the effectiveness, quality, and safety of various types of PPE, such as masks, gloves, gowns, and face shields. It aims to ensure that PPE used in Africa meets the necessary standards and offers optimal protection for health care workers and the general public during infectious disease outbreaks or other public health emergencies. The framework is being implemented through the establishment of testing facilities, training programs for laboratory personnel, and the dissemination of testing protocols. By establishing consistent testing procedures, the framework enhances the reliability and confidence in the performance of PPE and supports the procurement and distribution of high-quality equipment across African countries.^5^

### Framework for Regulation of Medical Devices and In Vitro Diagnostics

This framework provides a set of regulatory standards and requirements for the approval, registration, quality control, and post-market surveillance of medical devices and diagnostics. It aims to ensure that these products meet the necessary safety, efficacy, and quality standards and are appropriate for use in health care settings across Africa.^25^ The regulatory framework also establishes mechanisms for monitoring and assessing the performance and safety of medical devices and in vitro diagnostics, promoting transparency, and facilitating the harmonization of regulatory practices among African countries. By implementing this framework, Africa CDC aims to enhance the availability, accessibility, and reliability of medical devices, in vitro diagnostics, and vaccines to contribute to improved health care outcomes and public health in the region.^25^

### Framework for Action on Vaccine Manufacturing

The Framework for Action (FFA) answers the call made by the African Union Commission and the Africa CDC at a summit in April 2021 to develop a framework that will enable Africa to manufacture 60% of its vaccine needs locally by 2040. The fundamental idea behind the FFA is that Africa has the ability and should strive to establish a comprehensive interconnected system that stimulates investment in every stage of vaccine production, including research and development, drug substance manufacturing, and the final packaging process.^26^ The FFA advocates for expanding research and development efforts to encompass preclinical and clinical trials, with a specific focus on diseases that have significant consequences on the African continent. Additionally, the FFA suggests an increased emphasis on investment in the manufacturing of drug substances essential for vaccines that are crucial for Africa.^26^

## FUTURE AREAS FOR CAPACITY-BUILDING

### Upgrade Current Vaccine Capacity

At the moment, the vaccine supply for the African continent depends on the world market.^26^ Currently, vaccine manufacturers are based in only 5 African nations—South Africa, Morocco, Tunisia, Egypt, and Senegal. Research and development activities on the continent have been limited, with a narrow disease focus at the moment, hence justifying the need to upgrade. However, activity is increasing due to recent outbreaks like Ebola.^26^

African nations will need to invest in manufacturing capabilities to build a domestic vaccine manufacturing business. Such investments are not immediately appealing. The majority of equipment will need to be imported into Africa (especially equipment that is compliant with current good manufacturing practices), which will increase setup costs and subsequent revenue generation.

African nations will need to invest in manufacturing capabilities to build a domestic vaccine manufacturing business.

### Prioritize Diseases for Vaccine Production

The FFA designates the manufacture of vaccines for 22 diseases as a critical top priority using the integrated ecosystem strategy (Table).^26^ These include vaccines for 10 endemic or pandemic diseases for which vaccines are required, such as HIV, malaria, and COVID-19, which are typically high volume and can provide economies of scale; 6 expanding diseases, which are typically not yet commoditized vaccines or have relatively higher-priced vaccines; and 6 outbreak diseases, such as Ebola and Disease X (caused by a hypothetical, unknown pathogen and likely to result in an epidemic).^26^ By concentrating on these diseases, vaccinations that would be practical and desirable to produce might meet the urgent medical requirements of the continent.

**TABLE.** Twenty-Two Diseases Prioritized for the Africa CDC Framework for Action

Archetype	Disease	Vaccine Exists?	African Doses Volume by 2040, million	Disability-Adjusted Life Years 2040, million
**Legacy**: diseases with high coverage vaccines, primarily produced by Indian players at high volumes with low unit prices.	Hepatitis B, diphtheria, tetanus, whooping cough	Yes	∼370	6
	TB	Yes	∼140	12
	Measles	Yes	∼240	2
	Yellow fever	Yes	∼50	<1
	Cholera	Yes	∼30	1
	Typhoid	Yes	∼20	1
	Meningococcus^a^	Yes	∼60	5
**Expanding**: diseases that do not yet have commoditized vaccines, where vaccines are sold at relatively higher prices with some products still in development and not yet licensed.	Human papillomavirus	Yes	∼30	4
	Pneumococcus	Yes	∼140	13
	Rotavirus	Yes	∼120	9
	COVID-19	Yes	∼710	TBD
	Malaria	Yes	∼120	20
	HIV	No	∼110	10
**Outbreak**: diseases that are characterized by having emerging vaccines with unpredictable demand driven by outbreaks, often with higher prices due to lower scale and urgent need.	Ebola	Yes	∼1	9
	Influenza^b^	Yes	∼10	1
	Chikungunya	No	∼1	<1
	Rift Valley fever	No	∼1	<1
	Lassa fever	No	∼1	<1
	Disease X^c^	No	N/A	N/A
	Total		2,200	

Abbreviations: Africa CDC, Africa Centres for Disease Control and Prevention; N/A, not applicable; TBD, to be determined.

a Including key meningococcus serogroups found in Africa (A, C, W, and X).

b In this context, outbreak influenza.

c Disease X refers to a hypothetical, unknown pathogen that could emerge and result in an epidemic.

To meet the 2040 manufacturing target of 60% local production, it will also be essential to develop vaccines for 5 of the prioritized diseases for which no approved vaccine currently exists—HIV, chikungunya, Rift Valley fever, Lassa fever, and Disease X (upon emergence).^26^ The FFA also gives priority to 7 vaccine manufacturing methods to allow producers of these vaccines enough flexibility. These include both established technologies, like the production of live attenuated viruses, which will be essential in producing the high-demand vaccines, and cutting-edge ones, like mRNA, which are likely to expand in scope as the research and funding backing them develop. By supporting vaccine development for these priority diseases, pandemic preparedness is heightened, and the risk of zoonotic transmission of communicable diseases (e.g., Lassa fever) across international borders becomes significantly reduced.^26^ Furthermore, antimicrobial resistance becomes achievable through the development of therapeutics for these priority diseases.

### Use Criteria for Adopting Countries for Capacity-Building

#### Gavi Graduation

South Africa, Morocco, Tunisia, Egypt, and Senegal are currently involved in local vaccine production that meets approximately 1% of the total continental vaccine demand and should rank topmost in capacity-building for increased production.^26^ New or potential African manufacturers often find it challenging to access Gavi markets because of very competitive pricing that can only be achieved with significant economies of scale over time. By investing in existing manufacturers, challenges in market access can be overcome. Local manufacturers in these 5 countries are able to sell to Gavi-supported countries that can achieve volume certainty at scale.

#### National Regulatory Agencies

Presently, 92% of the African national regulatory agencies that have conducted the World Health Organization benchmarking using the global benchmarking tool are still at maturity level (ML)1, and only 2% have attained ML2.^26^ Most importantly, while Ghana and Tanzania are the only countries to have reached ML3, neither of them produces vaccines.^26^ Because Ghana and Tanzania have reached ML3, they should also be prioritized for capacity-building in vaccine production using the national regulatory agencies criterion. Both countries will require support with the national regulatory system, registration and marketing authorization, licensing of premises, market surveillance and control, vigilance, regulatory inspections, clinical trials oversight, laboratory access and testing, and lot release.

#### Demographics

In terms of demographics, Ethiopia (Eastern Africa), Nigeria (Western Africa), South Africa (Southern Africa), Egypt (Northern Africa), and the Democratic Republic of the Congo (Central Africa) should be prioritized. These 5 countries have the largest populations in Africa and should be adopted for capacity-building.

### Prioritize Point-of-Care Diagnostics

Point-of-care (POC) testing should be prioritized for both human and veterinary health sectors. POC testing refers to medical or diagnostic testing conducted near the patient or animal, allowing for rapid results and immediate decision-making.^27^^,^^28^ This approach offers several advantages, including quicker diagnosis, faster treatment initiation, and reduced reliance on centralized laboratory facilities. In the human health care sector, POC testing should be prioritized for various applications, including:

- **Infectious disease testing**. POC tests for infectious diseases, such as HIV, malaria, tuberculosis, or respiratory infections, can facilitate early diagnosis and prompt initiation of appropriate treatment. This is particularly important in African regions with limited access to health care facilities or remote areas where transportation of samples to central laboratories may be challenging.
- **Emergency medicine**. POC testing in emergency departments can expedite diagnosis and triage, leading to faster decision-making and appropriate patient management. For example, rapid tests for cardiac markers, such as troponin, can aid in the diagnosis of acute myocardial infarction, enabling timely interventions.
- **Chronic disease management**. POC testing devices can be used to monitor chronic diseases such as diabetes or hypertension. Patients can perform regular tests at home or in primary care settings, allowing for immediate feedback on their health status and adjustments in medication or lifestyle.

Point-of-care testing should be prioritized for both human and veterinary health sectors, as it offers quicker diagnosis, faster treatment initiation, and reduced reliance on centralized laboratory facilities.

In the veterinary sector, POC testing should be prioritized for:

- **Disease surveillance and outbreak management**. Rapid and on-site testing of animals, such as livestock or pets, can help in the early detection and control of infectious diseases, ensuring timely interventions to prevent further spread and minimize economic losses.
- **Veterinary emergency care**. POC testing in veterinary emergency situations can provide immediate diagnostic information, aiding in the prompt treatment of critical cases or injuries.
- **Livestock health monitoring**. POC testing can assist in the monitoring and control of diseases in livestock populations, enabling early detection and containment of outbreaks, as well as supporting decisions related to herd management and biosecurity measures.

### Manage the Strategic Reserve: Current and Future Strategies

To serve as a strategic reserve, the African Union Commission has designated 2 storage spaces with a combined area of 2,600 square meters at the AU Continental Logistics Base in Douala, Cameroon.^29^ The Africa CDC has implemented this strategic reserve with the aim of strengthening Africa's ability to prepare for and respond to health crises and outbreaks.^11^ The Africa CDC encourages member states to maintain strategic reserves to ensure the availability of essential medical resources, such as vaccines, therapeutics, and PPE, during outbreaks or public health emergencies.^11^ This facilitates a rapid response and prevents delays in obtaining crucial supplies. By establishing a strategic reserve, Africa reduces its reliance on external sources for medical provisions. This promotes self-sufficiency and reduces vulnerability to disruptions in the global supply chain, ensuring that African countries have access to essential health care resources when needed.

A strategic reserve promotes self-sufficiency and reduces vulnerability to disruptions in the global supply chain, ensuring that African countries have access to essential health care resources when needed.

The Africa CDC has played a role in facilitating the creation of strategic reserves by formulating guidelines to assist countries in establishing and overseeing such reserves. Moreover, capacity-building initiatives have been implemented to train national authorities and health care practitioners responsible for managing strategic reserves. These training programs encompass various areas, including inventory management, cold chain logistics, quality control, and distribution strategies, to ensure the efficient use of reserves. Furthermore, the Africa CDC has taken the lead in establishing regional stockpiles strategically positioned throughout the continent. These stockpiles function as regional centers for storing and distributing essential medical resources, thereby strengthening regional readiness and response capabilities.

#### Recommendations

The Africa CDC should collaborate with international entities responsible for managing global stockpiles, such as the World Health Organization's Contingency Fund for Emergencies and the Global Health Security Agenda. These global stockpiles can offer additional resources during public health emergencies. Similarly, regional organizations like the AU's AMSP should maintain their own regional stockpiles. The Africa CDC should work in coordination with these organizations to access and distribute supplies from these reserves whenever necessary.

The Africa CDC should conduct assessments and analyses to determine the optimal quantities of commodities required for the strategic reserve. These evaluations should consider various factors, including disease prevalence, population size, historical outbreak data, and resource availability. Regular reviews and updates should be carried out to ensure that the stockpile remains appropriate and relevant to the current situation. The Africa CDC should establish secure and suitable storage facilities to accommodate the strategic reserve, ensuring that different commodities are stored under the necessary conditions to maintain their quality and usability when needed. Distribution plans should also be devised, considering logistics, transportation, and the prioritization of resources to areas facing the most critical needs during emergencies.

To effectively manage the strategic reserve, Africa CDC should implement monitoring systems to track stock levels, expiration dates, and usage rates. Based on this information, regular replenishment can be carried out to ensure that the strategic reserve remains adequately stocked.

## CONCLUSION

With the right resources, support, and enabling environment, local manufacturers could successfully rise to the challenge of meeting Africa's PPE, vaccine, and diagnostic needs. Public-private partnerships will be critical to achieve this, which should include efforts by multilateral and regional institutions to patronize the production and supply from local manufacturers in line with international standards. Member states and their ministries of health must foster stronger collaborations with national regulatory agencies to share best practices and resources; harmonize standards; provide opportunities for post-marketing surveillance and flagging of substandard PPE, vaccines, and diagnostic equipment; and identify ways to enforce their standards to ensure safe, high-quality, and effective products are produced, procured, and used while adhering to international standard practices.

## Notes

**Peer Reviewed**

**First published online:** September 15, 2023.
